# Biomarkers of mitochondrial dysfunction in acute respiratory distress syndrome: A systematic review and meta-analysis

**DOI:** 10.3389/fmed.2022.1011819

**Published:** 2022-12-14

**Authors:** Catherine R. McClintock, Niamh Mulholland, Anna D. Krasnodembskaya

**Affiliations:** Wellcome-Wolfson Institute for Experimental Medicine, School of Medicine, Dentistry and Biomedical Sciences, Queen’s University Belfast, Belfast, United Kingdom

**Keywords:** acute respiratory distress syndrome, biomarker, mitochondrial dysfunction, mitochondrial DNA, mortality, systematic review, meta-analysis, ARDS

## Abstract

**Introduction:**

Acute respiratory distress syndrome (ARDS) is one of the main causes of Intensive Care Unit morbidity and mortality. Metabolic biomarkers of mitochondrial dysfunction are correlated with disease development and high mortality in many respiratory conditions, however it is not known if they can be used to assess risk of mortality in patients with ARDS.

**Objectives:**

The aim of this systematic review was to examine the link between recorded biomarkers of mitochondrial dysfunction in ARDS and mortality.

**Methods:**

A systematic review of CINAHL, EMBASE, MEDLINE, and Cochrane databases was performed. Studies had to include critically ill ARDS patients with reported biomarkers of mitochondrial dysfunction and mortality. Information on the levels of biomarkers reflective of energy metabolism and mitochondrial respiratory function, mitochondrial metabolites, coenzymes, and mitochondrial deoxyribonucleic acid (mtDNA) copy number was recorded. RevMan5.4 was used for meta-analysis. Biomarkers measured in the samples representative of systemic circulation were analyzed separately from the biomarkers measured in the samples representative of lung compartment. Cochrane risk of bias tool and Newcastle-Ottawa scale were used to evaluate publication bias (Prospero protocol: CRD42022288262).

**Results:**

Twenty-five studies were included in the systematic review and nine had raw data available for follow up meta-analysis. Biomarkers of mitochondrial dysfunction included mtDNA, glutathione coupled mediators, lactate, malondialdehyde, mitochondrial genetic defects, oxidative stress associated markers. Biomarkers that were eligible for meta-analysis inclusion were: xanthine, hypoxanthine, acetone, *N*-pentane, isoprene and mtDNA. Levels of mitochondrial biomarkers were significantly higher in ARDS than in non-ARDS controls (*P* = 0.0008) in the blood-based samples, whereas in the BAL the difference did not reach statistical significance (*P* = 0.14). mtDNA was the most frequently measured biomarker, its levels in the blood-based samples were significantly higher in ARDS compared to non-ARDS controls (*P* = 0.04). Difference between mtDNA levels in ARDS non-survivors compared to ARDS survivors did not reach statistical significance (*P* = 0.05).

**Conclusion:**

Increased levels of biomarkers of mitochondrial dysfunction in the blood-based samples are positively associated with ARDS. Circulating mtDNA is the most frequently measured biomarker of mitochondrial dysfunction, with significantly elevated levels in ARDS patients compared to non-ARDS controls. Its potential to predict risk of ARDS mortality requires further investigation.

**Systematic review registration:**

[https://www.crd.york.ac.uk/prospero], identifier [CRD42022288262].

## Introduction

Acute Respiratory Distress Syndrome is a principle cause of respiratory failure in critically ill patients requiring mechanical ventilation, characterized by severe pulmonary inflammation, diffuse alveolar damage and pulmonary edema (1). Due to a lack of effective treatment, ARDS results in substantial mortality of up to 30–40% (2). In addition to this, the current COVID-19 pandemic reports ARDS as one of the leading causes of ICU mortality, presenting an urgent need for advancement in ARDS research (3). ARDS pathogenesis remains nebulous; consequently, pharmacological therapies that reduce the severity of lung injury in preclinical models have not yet been translated into effective clinical treatment options. Therefore, further research into the mechanisms of ARDS pathogenesis and translational therapies is imperative.

Mitochondria are complex para-symbiotic organelles that perform a myriad of diverse yet interconnected functions, producing ATP and biosynthetic intermediates while also contributing to cellular stress responses such as autophagy and apoptosis (4). Acute inflammation can alter various mitochondrial functions, including reduced levels oxidative phosphorylation, and thus ATP production, increased mtROS production, increased apoptosis, as well as altered mitochondrial biogenesis and mitophagy (5). Dysfunctional mitochondria release multiple forms of damage-associated molecular patterns (DAMPs), such as ATP and mtDNA (6, 7). Similar to pathogenic stimuli, mitochondrial DAMPs can activate innate immunoreceptors, thus contributing to a vicious cycle of dysregulated inflammation. Other biochemical markers of mitochondrial dysfunction described in the literature include direct (lactate, pyruvate, lactate-to-pyruvate ratio, ubiquinone, alanine) and indirect markers (creatine kinase (CK), carnitine, aspartate aminotransferase (AST), alanine aminotransferase (ALT) and ammonia) (8). Clinical observational studies demonstrate that biochemical markers of mitochondrial dysfunction are associated with higher mortality and a higher risk of disease development in many respiratory conditions as well as sepsis (6, 9).

This review aims to assess the association between levels of biomarkers of mitochondrial dysfunction in any biological sample with mortality and other physiological and clinical outcomes in critically ill patients with ARDS. This will be carried out by presenting and appraising current research publications using standardized predefined assessable outcome measurements.

## Methods

This systematic review was conducted in accordance to the Preferred Reporting Items for Systematic Reviews and Meta-Analyses (PRISMA) and Cochrane guidelines. Please see Supplementary Methods 1 for extended explanations of search criterion.

### Literature search

The databases CINAHL, EMBASE, MEDLINE, and Cochrane were systematically searched using predefined search terms for headings: Mitochondria, ARDS, and patient; synonyms and analogous terms of these headlines were defined in Table 1. In order to be eligible studies must include adult (18y/o) participants with ARDS in intensive care units (ICU) (critically ill patients). The severity, cause, and duration of ARDS will not be restricted. The definition of ARDS was not a limiting factor. Covid-ARDS was not included in this systematic review due to differences in disease pathophysiology. No other exclusion criteria was applied to patients. Published relevant studies up to a March 5, 2022 were searched. Full search code, database limitation and limits applied for each database search can be found on PROSPERO (CRD42022288262).

**TABLE 1:** Title and abstract article screening terms.

Mitochondrial search terms (35 terms)	ARDS search terms (10 terms)	Patient search terms (8 terms)
Mitochondria	Acute respiratory distress syndrome	Patient
Mitochondrial function	ARDS	Case report
Mitochondrial dysfunction	Infant respiratory distress syndrome	Case study
Mitochondrial disorder	Infantile respiratory distress syndrome	Human subject
Mitochondrial respiration	IRDS	Human
Mitochondrial biogenesis	Adult respiratory distress syndrome	Trial
Mitochondrial homeostasis	Respiratory distress syndrome adult	Human trial
Mitochondrial fitness	Respiratory insufficiency	Clinical trial
Mitochondrial DNA	Respiratory failure	
mtDNA	Respiratory distress syndrome	
Cytopathic hypoxia		
Mitochondrial RNA		
Mitochondrial miRNA		
mitoMIRs		
Aerobic metabolism		
ATP		
Adenosine triphosphate		
Tricarboxylic acid cycle		
TCA cycle		
Krebs cycle		
Electron transport chain		
ROS		
Reactive oxygen species		
Oxidative stress		
OXPHOS		
Oxidative phosphorylation		
Retrograde signaling		
Sirtuins		
SIRT		
Amino acid synthesis		
Fatty acid oxidation		
Mitophagy		
Biogenesis		
Fission		
Fusion		

### Study selection

Following the initial procurement of studies, by McClintock and Mulholland independently, from search databases, articles were retrieved in full text and stored on Endnote software. A 97% similarity in search results was obtained upon comparison of independent searches. Endnote enabled removal of duplicate articles. Those studies initially applicable were reviewed in full and criterion assessed, those failing to meet criterion were omitted.

### Data extraction

The primary outcome was to assess the association between levels of biomarkers of mitochondrial dysfunction in clinical samples and ARDS patient mortality. The secondary outcome was to assess the association between levels of biomarkers of mitochondrial dysfunction in clinical samples and; (i) disease development and (ii) aggravation of ARDS disease severity. Alongside outcome data, the following information was also extracted: patient characteristics (age and sex), year of study publication, study design, sample size, characteristics of ARDS, type of biomarker, type of clinical sample, time of sampling, methods of biomarker measurement, concentration levels of the biomarkers.

All study designs were eligible for inclusion in this systematic review. Studies lacking a comparator/control, for example in the instance of retrospective case reports, were not eligible for meta-analysis inclusion. In the case of interventional studies, the data were extracted from the non-interventional/control arm of the study; this was to ensure that the mitochondrial biomarkers were not confounded by the intervention carried out.

Where possible biomarker concentration data for meta-analysis was collected in the form of mean, standardized mean difference (SMD) and “*N*” study participant. In the cases where median with interquartile range (IQR) were the only data provided, they were converted to Mean ± SD using the range rule (the standard deviation of a sample is approximately equal to one-fourth of the range of the data). Any standard errors provided were converted to standard deviation for consistency. RevMan software 5.4 was used to store and analyze data, as recommend by Cochrane guidelines. The random-effects model using the inverse- variance method was used on this statistical software, as this allowed for studies with the same biomarker lacking the same units to be compared. The *I*^2^ statistics was used to analyze between-study heterogeneity, and values higher than 50% was considered as high heterogeneity. *P*-values less than 0.05 were considered significant.

### Quality assessment

Given the inclusion of all study designs in this systematic review, the risk of bias assessment methods used to assess study quality were chosen based on applicable nature to study design. The study design was confirmed using the SIGN checklist prior to assessmen.^1^ Randomized Control Trials (RCT) were grouped together and assessed using the Cochrane risk of bias tool^2^ and non-randomized studies, were assessed using the Newcastle-Ottawa Scale (NOS).^3^ Studies were considered high quality if the NOS score was more than six points.

## Results

### Study selection

**FIGURE 1:**
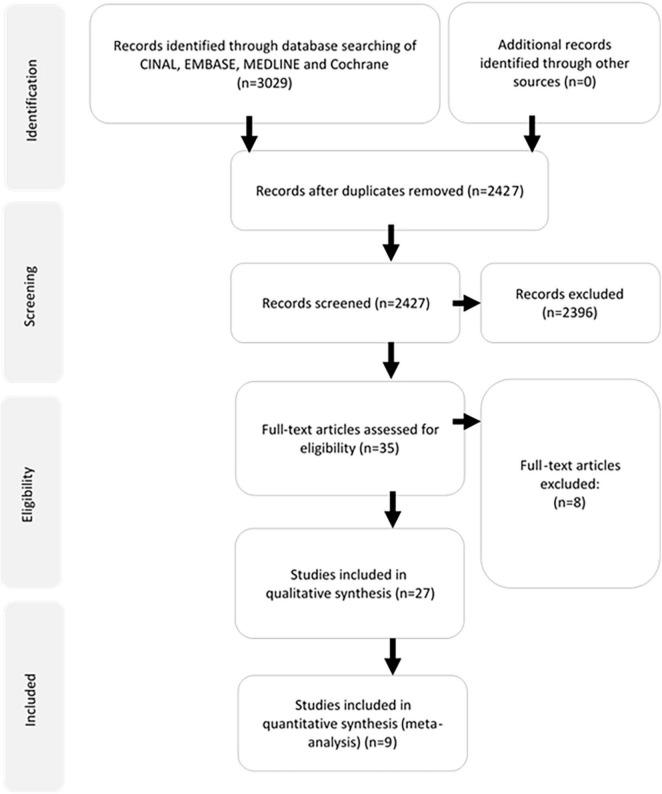
PRISMA flow diagram of literature search for studies included in this review.

A total of 3,029 articles were identified through search of the four databases. Twenty-six articles were included in the review, nine of which contained sufficient information for meta-analysis. The selection process has been summarized according to the PRISMA guidelines in Figure 1. No potentially relevant papers were excluded from review.

### Patient and study characteristics

Participant characteristics are summarized in the Table 2. The mean number of ARDS patients across all included articles was 79 and the mean non-ARDS was 148. In the ARDS studies there was a greater number of male participants (53.9% male), than in the non-ARDS (52.8% male). The mean ages of the ARDS participants was 55.4 and non-ARDS 51.3. The type and cause of ARDS varied, in the studies that provided this information, pneumonia and sepsis were the most prevalent causes of ARDS. There was a degree in variation of sample collection time, the majority of samples were collected upon enrollment or day 0 (52%) and the maximal collection time was 35 days. 29 sample sources across 25 studies. Sample sources were: blood (34.4%), plasma (34.4%), BAL (27.6%) and muscle tissue (3.45%).

**TABLE 2:** Summary of patient characteristics.

References	Patient sample size		Patient age		Patient sex		ARDS characteristic type/Cause
	ARDS	Non-ARDS	ARDS	Non-ARDS	ARDS male	Non-ARDS male	
Quinlan et al. (29)	29	6	35.9 ± 18	NR	14 (48.3)	NR	Trauma (24.1), pneumonia (10.3), sepsis (10.3), aspiration (10.3) and other (55.3)
Ortolani et al. (17)	12	0	55 ± 13	0 (0)	7 (58.3)	0 (0)	NR
Nathens et al. (30)	0	294	0	39 ± 15	0	222 (76)	NR
Scholpp et al. (24)	13	10	43.2 ± 12	44.1 ± 18	4 (5.3)	50 (66.7)	Pneumonia (30.7), sepsis (7.7) and other (61.6)
Nelson et al. (31)	94	62	NR	41 ± 1.22	NR	NR	NR
Soltan-Sharifi et al. (18)	10	0	52.7 ± 7.2	0	NR	0	NR
Moradi et al. (19)	13	0	49.2 ± 4.5	0	8 (61.5)	0	NR
Nakahira et al. (10)	134	309	49.5 ± ≈12.5*	275 (62.1)	NR		
Bhargava et al. (26)	22	0	49.4 ± ≈15.28*	0	16 (72.7)	0	Sepsis (27), pneumonia (59) and other (14)
Evans et al. (20)	18	8	46.1 ± 14.9	39.8 ± 11.0	9.9 (55)	4 (50)	Sepsis (39), aspiration (22), pneumonia (33) and other/unknown (5)
Liu et al. (25)	18	10	58.34 ± 8.25	57.93 ± 7.96	12 (66.7)	6 (60)	NR
Serpa et al. (22)	545	0	41.4 ± 14	0	331 (60.7)	0	Type: Pulmonary (92.4) and non-pulmonary (7.6) Cause: Pnemonia (83.8), non-pulmonary sepsis (1.8), trauma (8.9) and other (5.5)
Dorward et al. (27)	12 Divided into: 10 BAL and 7 serum sample for exp1 3 BAL and 6 serum samples for exp2	10 Divided into: 10 BAL and 8 serum samples 3 BAL and 6 serum samples	58 ± ≈30.5*	60 ± ≈30*	Calculation not available (64)	16.69 (79)	NR
Garramone et al. (11)	60	0	76.9 ± 13.0	0	34.2 (57)	0	NR
Fredenburgh et al. (33)	Cohort 1: 2 Cohort 2: 2	0	Cohort 1: 57 ± 19, Cohort 2: 49 ± 9	0 (0)	NR	0 (0)	NR
Mahmoodpoor et al. (21)	20	0	58 ± ≈10.5*	0 (0)	11 (55)	0 (0)	ARDS with comorbidities Sepsis (20) Surgery/Trauma (25) pneumonia (10)
Grazioli et al. (13)	8	3	NR	NR	NR	NR	NR
Bos et al. (28)	Phenotype one: 82 (uninflamed) ARDS with sepsis Phenotype two: 128 (reactive) ARDS with sepsis	547 sepsis 42 healthy control	64 ± ≈8*	62.258 ± ≈12.6*	121 (58)	323 (59)	Sepsis ARDS
Rosenberg et al. (32)	142	0	65 ± 0	0 (0)	64 (45.1)	0 (0)	NR
Blot et al. (14)	7 ARDS	14	60.5	50 (32–54 IQR)	17 (80.9)	5 (17.9)	NR
Huang et al. (15)	73	0	64	0	57 (78.1)	0 (0)	Pneumonia (57.53), aspiration (10/96), trauma (6.85), drowning (2.74), sepsis (16.44) and other (5.48)
Faust et al. (6)	PETROS: 41 MESSI: 45	PETROS: 183 MESSI: 75	44 ± 15≈* PETROS 62 ± 7.5≈* MESSI	33 ± 15≈18* PETROS 60 ± 18≈* MESSI	52 (15.1)	176 (51.2)	Trauma and sepsis
Korsunov et al. (23)	14	15	68 ± ≈7*	60.5 ± ≈14.25*	NR	NR	NR
Hernandez-Beeftink et al. (16)	264	423	63 ± 14	64 ± 15	175 (66)	255 (60)	Sepsis (100)

NR, not recorded; ARDS, acute respiratory distress syndrome; BAL, broncho-alveolar lavage; PETROS, Penn trauma organ dysfunction study; MESSI, molecular epidemiology of sepsis in the ICU; ICU, intensive care unit.

*Approximate range rule calculations.

Biomarkers of mitochondrial dysfunction reported in the included studies were: mitochondrial DNA (mtDNA) (eight studies) (6, 10–16) glutathione coupled mediators [(referenced retrospectively, glutathione, glutanation, glutathione *S*-transferase (GST), L-gluatamate and glutathione perosidase)] (five studies) (17–21), lactate (three studies) (20, 22, 23), malondialdehyde (MDA) (three studies) (17, 24, 25), metabolic signaling pathways and mediators [(referenced retrospectively: Nicotinamide adenine dinucleotide (NADH), N-terminal peptide FMNPLAQ - also known as NADH2, glyceraldehyde-3-phosphate dehydrogenase-like 6 (GAPDHL6), sirtuin enrichment, xanthine and hypoxanthine)] (four studies) (26–29), oxidative stress associated markers [(hydrogen peroxide (H_2_O_2_), super oxidase dismutase (SOD), ascorbate, alpha tocopherol, beta-carotene and retinol] (three studies) (24, 30, 31) (Table 3). Notably, multiple studies reported more than one biomarker, as recorded in Tables 2–5. Methods of sample analysis varied depending on the nature of sample biomarker. Mitochondrial DNA was recurrently analyzed using PCR (based on mitochondrial copy number|), other markers were analyzed using HPLC, ELISA, enzyme immunoassay, mass spectrometry and GeneTitan Affymetrix (Table 3).

**TABLE 3:** Summary of study characteristics.

References	Study design	Sample size		Sample source	Sample moment	Sample analysis	Biomarker of mitochondrial dysfunction
		ARDS	Non-ARDS				
Quinlan et al. (29)	Observational study	29	6	Plasma and BAL	Plasma: 24 h after closure of venous catheter BAL: admission into ICU (from 11 ARDS patients)	HPLC analysis	Xanthine and hypoxanthine
Ortolani et al. (17)	RCT	12	0	BAL	Days 0,3,6 and 9 of placebo therapy	HPLC analysis	Glutathione and malondialdehyde (MDA)
Nathens et al. (30)	RCT	0	294	Plasma and BAL	Days 1,3,5,7,14 and 21 after admission	Enzyme immunoassay	Ascorbate and alpha tocopherol (metabolites)
Scholpp et al. (24)	Observational study	13	10	Plasma	Second day after admission to the intensive care unit (ICU)	HPLC analysis	Lipid peroxidation markers (acetone, isoprene and *n*-pentane) and MDA
Nelson et al. (31)	Permuted block randomized, single blinded trial	94	62	Plasma	Upon enrollment to study	HPLC analysis	Lipid peroxidation markers (beta-carotene, retinol, and α- tocopherol)
Soltan-Sharifi et al. (18)	Randomized interventional trial	10	0	Blood – red blood cells	(time 0), and times 24, 48, and 72 h post administration	GSH assay	Glutation (GSH) and *N*-acetylcysteine
Moradi et al. (9)	Prospective randomized single blinded trial	13	0	Peripheral blood	Administration of placebo day 0, samples taken day 2, 3, and 4	DNA genotyping via PCR	Three glutathione-*S*-transferase (GST) isoforms: GST m1, GST T1, GST P1
Nakahira et al. (10)	Retrospective study with two cohorts	134	309	Plasma	Upon initial enrollment of patients	qPCR	mtDNA copy number (NADH dehydrogenase 1 DNA level
Bhargava et al. (26)	Exploratory patient sample study	22	0	BAL	(Day 1–7) or the late phase (day 8–35)	iTRAQ labeling and 2D LC-Orbitrap M	Glycolysis protein expression and enrichment
Evans et al. (20)	Pre- RCT study	18	8	BAL	0–72 h of the diagnosis of ARDS	Chromatographic method	Metabolite ion chromatographs (L-glutamate, hypoxanthine, xanthine and L-lactate)
Liu et al. (25)	Case-control study	18	10	Arterial blood serum	T1,T2,T3, and T4	Assays for mediators of inflammation and oxidative stress	MDA, superoxide dismutase (SOD) and hydrogen peroxide (H_2_O_2_)
Serpa et al.(22)	Meta-analysis of observational studies	545	0	Arterial blood	Upon initial enrollment of patients	NR	Lactate measurement
Dorward et al. (27)	Retrospective study	12 Divided into: 10 BAL and 7 serum sample for exp1 3 BAL and 6 serum samples for exp2	10 Divided into: 10 BAL and 8 serum samples 3 BAL and 6 serum samples	BAL and blood	Upon initial enrollment of patients	Exp1: Liquid chromatography– tandem mass spectrometry Exp2: qPCR	Exp1: *N*-Formylated mitochondrial peptides: (*N*-formylated termini of NADH-ubiquinone oxidoreductase chain 2 (NADH2; fMNPLAQ) and NADH-ubiquinone oxidoreductase chain 4 L (NADH4L; fMPLIYM) Exp2: mtDNA
Garramone et al. (11)	Cohort study	60	0	Blood plasma and serum	Upon initial enrollment of patients	ELIZA	Soluble Nox2-derived peptide (sNOX2-dp) a marker of NADPH-oxidase activity
Fredenburgh et al. (33)	Interventional double-blinded randomized parallel assigned trial	Cohort 1: 2 Cohort 2: 2	0	Plasma	Prior to treatment on day 1 and after treatment on days 1–5 and 7	Quantitative PCR of human NADH dehydrogenase 1 (MTND1)	mtDNA
Mahmoodpoor et al. (21)	Double-blind placebo-controlled randomized parallel clinical trial	20	0	Blood	Day 0,day 7, and day 14	Enzyme-linked immunosorent assay (EILZA)	Natural levels of selenium (glutathione peroxidase)
Pan et al. (12)	Case report	1	0	Muscle tissue	Upon admission to ICU	PCR	mtDNA
Grazioli et al. (13)	Observational	8	3	BAL	Day 1 and day 7	qRT-PCR	mtDNA
Bos et al. (28)	Observational prospective study	Phenotype one: 82 (uninflamed) ARDS with sepsis phenotype two: 128 (reactive) ARDS with sepsis	547 sepsis 42 healthy control	Whole blood	Within 24 h of ICU admission	Human genome U219 96-array plates and the GeneTitan instrument (Affymetrix)	mRNA
Rosenberg et al. (32)	Retrospective preliminary study	142	0	Blood	One week prior to hospital discharge	ELIZA	GDF-15
Blot et al. (14)	Observational case-control prospective study	7 ARDS	14	BAL and plasma	Upon enrollment	qPCR	mtDNA
Huang et al. (15)	Observational study	73	0	Plasma	Days 1, 3, and 7 after ICU admission	RT-qPCR	mtDNA
Faust et al. (6)	Two sided prospective study	41 PETROS cohort (trauma patients) 45 MESSI cohort (sepsis patients)	183 PETROS 75 MESSI	Plasma	At ED presentation and 48 h later	PCR	mtDNA
Korsunov et al. (23)	Single-center prospective comparative study	14	15	Arterial blood	Taken upon enrollment to study, over the period July–October 2021	Lactate = Chemray 120 Mindray biochemical analyser (China)	Lactate and oxygen transport
Hernandez-Beeftink et al. (16)	National, multicenter, observational study	264	423	Peripheral blood	24 h of sepsis diagnosis	mtDNA probes from the array data – CEU 1 array data	mtDNA

NR, not recorded; BAL, broncho-alveolar lavage; ICU, intensive care unit; HPLC, high-performance liquid chromatographic; RCT, randomized controlled trial; MDA, malondialdehyde; PCR, polymerase chain reaction; SNP, single nucleotide polymorphism; NAC, *N*-acetylcysteine; GST, glutathione-*S*-transferase; BWH RoCI, Brigham and Women’s Hospital registry of critical illness; ME ARDS, molecular epidemiology of acute respiratory distress syndrome; mtDNA, mitochondrial deoxyribonucleic acid; ED, emergency department; ELISA, enzyme-linked immunosorbent assay; NIV, non-invasive ventilation; sNOX2-dp, Nox2-derived peptide; ARF, acute respiratory failure; NADPH, nicotinamide adenine dinucleotide phosphate; iCO, inhaled carbon monoxide; PETROS, Penn trauma organ dysfunction study; MESSI, molecular epidemiology of sepsis in the ICU; qRT-PCR, real-time quantitative reverse transcription; tRNA, transfer ribonucleic acid; GDF-15, growth differentiation factor-15; RCT, randomized controlled trial; mtDNA, mitochondrial deoxyribonucleic acid; MDA, malondialdehyde.

**TABLE 4:** Primary outcome: Association of mitochondrial biomarker levels with ARDS mortality.

References	Biomarker	Mortality rates non-survivors		Biomarker summary		Summary of statistical comparison with mortality	Mortality time point	Association conclusion
		ARDS	Non-ARDS	ARDS	Non-ARDS			
Quinlan et al. (29)	Hypoxanthine and xanthine	14 (48.3)	0 (0)	Plasma Xanthine: *S* = 13.3 ± 2.01 NS = 7.76 ± 0.09 Plasma Hypoxanthine: *S* = (15.24 ± 2.09) NS = (37.48 ± 3.1)	Plasma Xanthine: (9.4 ± 2.7) Plasma Hypoxanthine: (1.69 ± 0.76)	Plasma Xanthine: S vs NS *P* = 0.68 ARDS vs non = *P* > 0.05 Plasma Hypoxanthine: S vs NS *P* = 0.001 ARDS vs Non *P* < 0.01 No association with BAL	Time point unrecorded	Possible correlation Significant association No association
Ortolani et al. (17)	Glutathione and Malondialdehyde (MDA)	7 (58.3)	0 (0)	Glutathione (<450 μM to <550 nM) Malondialdehyde (<4 to >4 nM)	0	Both markers show non-significant positive association	28-day	Possible correlation
Nathens et al. (30)	Ascorbate and alpha tocopherol	0 (0) 0 (0)	7 (2.4) 9 (3.1) 9 (3.1)	Not applicable	Ascorbate: day-0 ≤ 0.5, day-21 ≤ 0.5 Alpha-tocopherol: day- <5, day-21 ≥ 10	53 (18) patients in non-ARDS cohort developed ARDS, statistics not carried out	28-day ICU Hospital	Possible correlation
Scholpp et al. (24)	MDA and lipid peroxidation markers (acetone, isoprene and pentane)	NR	NR	MDA: 0.55 Acetone: 1.32 Isoprene: 50.0 *n*-Petane: 1	MDA: 0.38 Acetone: 0.55 Isoprene: 33.2 *n*-Petane: 0.12	*n*-Petane statistically different in ARDS vs NON-ARDS	NR	Not applicable to this study
Nelson et al. (31)	Lipid peroxidation markers (beta-carotene, retinol, and α- tocopherol)	NR	NR	Exact values not provided: Beta carotene, retinol and α- tocopherol all significantly reduced in ARDS vs NON-ARDS			NR	Not applicable to this study
Soltan-Sharifi et al. (18)	Glutation (GSH) and *N*-acetylcysteine	NR	NR	GSH 0 h – <600 increased to <800 at 72 h	0	Not calculated, no trend of association with disease and time	NR	Not applicable to this study
Moradi et al. (19)	GST isoforms: M1, T1 and P1	10 (76.9)	0 (0)	Significant association of mortality with GST M1 null polymorphism and double deletion of both genes (M1, T1) in control ARDS placebo group of interest (*P* < 0.05). No significance for the GST P1 isoform with mortality. Absence of the GST M1 gene/deletion of both GTS M1 and GST T1 are more vulnerable to oxidative stress contributing to ARDS/ALI		Mortality with GST M1 *P* < 0.05 T1 and P1 non-significant	NR	Significant association
Nakahira et al. (10)	mtDNA copy number (NADH dehydrogenase 1 DNA level)	BWH 60 (30) ME 40 (16)		BWH [46,648 (14,468–63,510)] ME [29,828 (7,857–84,675)]	BWH [10,584 (3,992–41,466)] ME [8,771 (3,296–20,464)]	Only median and IQR provided – unable to calculate	28-day	Unclear correlation
Bhargava et al. (26)	Glycolysis protein expression & enrichment, and thioredoxin	15 (68.2)	0 (0)	Glycolysis is enriched in ARDS non-survivors with a fold change increase of 2.01 for GAPDHL6 (an example gene from the list of glycolytic proteins). This was not enriched in survivors. thioredoxin: *S* = apx. 2.5 NS = apx. 7.5	0	Significance was not provide with fold change thioredoxin *P* < 0.05	NR	Possible correlation Significant association
Evans et al. (20)	Metabolite ion chromatographs (L-glutamate, hypoxanthine, xanthine and L-lactate)	NR	NR	Metabolite fold change expression of ARDS vs healthy controls: L-Glutamate 7.94FC Hypoxanthine 40.96FC L-Lactate 3.49FC		L-Glutamate *P* = 2.49E-06 Hypoxanthine *P* = 6.93E-10 L-Lactate *P* = 0.0437	NR	Not applicable to this study
Liu et al. (25)	MDA, superoxide dismutase (SOD) and hydrogen peroxide (H_2_O_2_)	0 (0) Two patient mortality recorded – unclear as to which group		MDA = significantly higher in ARDS group (*P* = 0.01) SOD = significantly lower in ARDS group (*P* < 0.01) H_2_O_2_ = significantly higher in ARDS group (*P* = 0.02)			4-day One year follow up	Unclear findings
Serpa et al. (22)	Lactate measurement	192 (35.23)	0 (0)	Lactate: *S* = (29.9 ± 34.8) NS = (46.7 ± 43.0)	0	*P* = 0.003 Significant increase in lactate in non-survivors of ARDs	28-day	Significant association
Dorward et al. (27)	NADH, FMNPLAQ and NADH-	5 (42)	NR	Exact numbers not provided in text. Mitochondrial formylated peptides were elevated in BAL and serum from patients with ARDS. Bal and serum showed strong significant increase in markers in ARDS patients to healthy patient controls (*P* < 0.001) (Exp1). mtDNA recorded in BAL (*n* = 3 per group) and serum (*n* = 6 per group) showed *p* < 0.05 increase in mtDNA copy number in ARDS group compared to healthy control (Exp2).			NR	Unclear
Fredenburgh et al. (33)	mtDNA	NR	NR	Initial enrollment level: 7,218.0 End of study: 24,083.5	NR	Non-significant increase between biomarker levels, mortality not included in study	NR	Not applicable to this study
Mahmoodpoor et al. (21)	Natural levels of selenium (glutathione peroxidase)	16 (22.2)	0 (0)	Sele-: Day 0 – apx. > 75 Day 14 – apx > 75 Exact data values not available	0	Non-significant	14- day	Non-significant positive association
Pan et al. (12)	mtDNA	NR	NR	Patient was diagnosed with mitochondrial myopathy. Pathological findings of RRF in a muscle biopsy and genetic analysis of an A3243G point mutation in the tRNALEU (UUR) gene of mtDNA. Paper indicates a link between respiratory failure and mtDNA mutation in adult.			NR	Not applicable to this study
Grazioli et al. (13)	mtDNA	NR	NR	179.8583777	1.0	Not calculated	NR	Not applicable to this study
Bos et al. (28)	Mitochondrial canonical pathways based off mRNA expression	59 (28)	95 (17.4)	Pathway expression levels of mitochondrial function: SIRTUIN signaling and oxidative phosphorylation. ARDS fold change compared to Non-ARDS and significance presented in paper.		Statistical testing *P* < 0.001 Significant difference between mitochondrial dysfunctional genes/SIRTUIN pathway, oxidative phosphorylation in sepsis ARDS compared to sepsis. Mortality statistics not calculated.	60-day	Possible correlation
Rosenberg et al. (32)	GDF15	21 (14.8)	0 (0)	Raised levels of GDF-15 prior to discharge, and lower in recovery	NR	Not calculated	NR	Unclear
Blot et al. (14)	mtDNA	1 (14)	NR	0.1503	0.01546	No calculation available	30 day	Unclear
Huang et al. (15)	mtDNA	36 (49.3)	0 (0)	Severe: 1,230 (588–22,387) Moderate: 5,370 (628–13,052) Mild: 15,792 (1,623–186,814) *S*: 7,585 (1,717–15,792) NS: 67,608 (19,498–346,736)	NR	Severe ARDS vs Mild ARDS mtDNA levels *P* = 0.03 *P* < 0.05 – higher levels of mtDNA in ARDS survivors vs ARDS non-survivors	Day-7	Significant association
Faust et al. (6)	mtDNA	PETROS: 17 (7.6) MESSI: 50 (41.7)		PETROS: ARDS = 12.28 1.07 MESSI: ARDS = 11.06 1.31	PETROS: NON = 12.04 1.01 MESSI: NON = 11.25 1.20	PETROS: ARDS vs NON *P* = 0.009 S vs NS *P* = 0.06 MESSI: ARDS vs NON *P* = 0.003 S vs NS *P* = 0.073	30-day	Significant association
Korsunov et al. (23)	Lactate and oxygen transport	14 (100)	11 (73.3)	3.4 ± 3.75	5.3 ± 0.675	Not carried out, correlative trend evident in ARDS vs NON-ARDS	NR	Possible correlation
Hernandez-Beeftink et al. (16)	mtDNA	39 (82)	45 (174)	3.65 (1.39–9.59 (hazard ratio and 95% CL) 0.031 ± 0.2036 *S*: –0.0038 ± 0.2012 NS: 0.0702 ± 0.2001	1.24 (0.44–3.51) –0.0073 ± 0.2004	Non-ARDS *P* = 0.683 ARDS *P* = 0.009 mtDNA significantly associated with 28-day mortality	28-day	Significant association

S, survivor; NS, non-survivor; NR, not recorded; CL, confidence limit.

**TABLE 5:** Secondary outcome: Association between levels of mitochondrial biomarkers and risks of development of new complications of ARDS or worsening of severity or development of ARDS.

References	Biomarker	Biomarker summary		Association to disease presence/ progression	Association to other worse outcomes of ARDS
		ARDS	Non-ARDS		
Quinlan et al. (29)	Hypoxanthine and xanthine	Plasma xanthine: *S* = 13.3 ± 2.01 NS = 7.76 ± 0.09 Plasma hypoxanthine: *S* = (15.24 ± 2.09) NS = (37.48 ± 3.1)	Plasma xanthine: (9.4 ± 2.7) Plasma hypoxanthine: (1.69 ± 0.76)	Significant association with both hypoxanthine (Plasma *P* < 0.01 and BAL *P* < 0.02). Xanthine (plasma *P* < 0.05 and BAL *P* < 0.01)	NR
Ortolani et al. (17)	Glutathione and malondialdehyde (MDA)	Glutathione (<450 μM to <550 nM) Malondialdehyde (<4 to>4 nM)	0	Non-significant trend increase with time (9 days) of both Glutathione and Malondialdehyde	NR
Nathens et al. (30)	Ascorbate and alpha tocopherol	Not applicable	Ascorbate: day-0 ≤ 0.5, day-21 ≤ 0.5 Alpha-tocopherol: day- < 5, day-21 ≥ 10	Non-significant positive association (18% developed ARDS)	Multiple organ failure in 6.1% of subjects. Pneumonia in 15% at 28-day follow up, and 1.3% had renal failure
Scholpp et al. (24)	MDA and lipid peroxidation markers (acetone, isoprene and pentane)	MDA: 0.55 Acetone: 1.32 Isoprene: 50.0 *n*-Petane: 1	MDA: 0.38 Acetone: 0.55 Isoprene: 33.2 *n*-Petane: 0.12	Significant positive association for *N*-pentane (*P* < 0.05) non-significant positive association for MDA acetone and isoprene	NR
Nelson et al. (31)	Lipid peroxidation markers (beta-carotene, retinol, and α- tocopherol)	Exact values not provided: Beta carotene, retinol and α- tocopherol all significantly reduced in ARDS vs NON-ARDS		Significant levels of lipid peroxidation markers in ARDS vs non-ARDS patients (*P* > 0.05)	NR
Soltan-Sharifi et al. (18)	Glutation (GSH) and *N*-acetylcysteine	GSH 0 h – <600 increased to <800 at 72 h	0	Non-significant trend of association with time (3-days) with ARDS disease	NR
Moradi et al. (19)	GST isoforms: M1, T1 and P1	Significant association of mortality with GST M1 null polymorphism and double deletion of both genes (M1, T1) in control ARDS placebo group of interest (*P* < 0.05). No significance for the GST P1 isoform with mortality. Absence of the GST M1 gene/deletion of both GTS M1 and GST T1 are more vulnerable to oxidative stress contributing to ARDS/ALI		No association with recorded factors of duration of mechanical ventilation or Length of ICU stay Not directly applicable (NR)	NR
Nakahira et al. (10)	mtDNA copy number (NADH dehydrogenase 1 DNA level	BWH [46,648 (14,468–63,510)] ME [29,828 (7,857–84,675)]	BWH [10,584 (3,992–41,466)] ME [8,771 (3,296–20,464)]	Non-significant trend in association	NR
Bhargava et al. (26)	Glycolysis protein expression & enrichment, and Thioredoxin	Glycolysis is enriched in ARDS non-survivors with a fold change increase of 2.01 for GAPDHL6 (an example gene from the list of glycolytic proteins). This was not enriched in survivors Thioredoxin: *S* = apx. 2.5 NS = apx. 7.5	0	NR	NR
Rosenberg et al. (32)	GDF15	Raised levels of GDF-15 prior to discharge, and lower in recovery	NR	NR	More comorbidities present in higher GDF-15 quartiles – non-significant association
Blot et al. (14)	mtDNA	0.1503	0.01546	mtDNA levels significantly raised in ARDS patients indicating association significant *P* = 0.02	NR
Huang et al. (15)	mtDNA	Severe: 1,230 (588–22,387) Moderate: 5,370 (628–13,052) Mild: 15,792 (1,623–186,814) *S*: 7,585 (1,717–15,792) NS: 67,608 (19,498–346,736)	NR	Significant association *p* = 0.04	NR
Faust et al. (6)	mtDNA	PETROS: ARDS = 12.28 1.07 MESSI: ARDS = 11.06 1.31	PETROS: NON = 12.04 1.01 MESSI: NON = 11.25 1.20	Significant positive association (*P* = 0.009) Pero S (*P* = 0.003)	NR
Korsunov et al. (23)	Lactate and oxygen transport	3.4	5.3	Moderate evidence to suggest lower levels of lactate in ARDS patients	In-group one AKI was diagnosed in 8 patients (57.14%) which is twice as much as group 2–4 (26.7%)
Hernández-Beeftink et al. (16)	mtDNA	3.65 (1.39–9.59 (hazard ratio and 95% CL) 0.031 ± 0.2036 *S*: –0.0038 ± 0.2012 NS: 0.0702 ± 0.2001	1.24 (0.44–3.51) –0.0073 ± 0.2004	Non-significant trend of association	NR

S, survivor; NS, non-survivor; NR, not recorded; CL, confidence limit.

Mortality was recorded in seventeen of the twenty-five studies included in this review (Table 4). Mortality time point recording ranged from 4 to 60-day; however a number of studies failed to record a specific time point of mortality used in the analysis. Studies were examined for significant associations between raised biomarker levels and mortality outcomes, as well as non-significant trends. Due to a number of studies reporting multiple biomarkers, two studies fell into both of these categories (27, 30).

In the 17 studies which had recorded mortality, seven (41%) reported a significant association between elevated levels of mitochondrial biomarkers and higher mortality of ARDS patients (6, 15, 16, 19, 22, 27, 30). From these studies the significantly associated biomarkers were: hypoxanthine (30), GST isoform M1 (19), Thioredoxin (27), lactate (22) and mtDNA (6, 14–16). Due to the lack of provided numerical information only three studies were eligible for inclusion into the meta-analysis for association with mortality. Mitochondrial DNA was the only biomarker reported with significant association in more than one study.

Seven studies (41%) reported numerical trends toward association of higher levels of mitochondrial biomarkers with higher mortality, however the association did not reach statistical significance, or insufficient numerical information was provided (17, 23, 27, 29–32). Positively but non-significantly associated with mortality biomarkers consisted of: xanthine (30), glutathione, MDA (17), ascorbate, alpha-tocopherol (31), GAPDHL6 (27), glutathione peroxidase (selenium) (21), mediators of sirtuin signaling pathway, mediators of oxidative phosphorylation (29) and lactate (23).

The five (29%) remaining study biomarkers, from the 17 eligible for mortality association assessment, did not show any significant, nor general trend with biomarker levels and mortality (10, 14, 25, 27, 32). Of these five studies, two reported mortality of ARDS and non-ARDS groups together (10, 25), and two did not report mortality in non-ARDS group (14, 27), due to inability to draw comparisons between ARDS and non-ARDS cohorts for these four studies, no conclusions of association could be drawn. The remaining study has unclear findings. In Rosenberg et al., raised levels of GDF-19 were found in patients prior to hospital discharge, before declining in recovery. Whilst patient mortality was recorded, due to the temporary nature of increased biomarker levels no conclusive association can be drawn (33).

Development of other adverse clinical outcomes was recorded in five studies. These clinical outcomes included: multiple organ failure, renal failure, pulmonary fibrosis, atrial thrombus, hypotension and acute kidney injury. No statistical correlations of measured levels of mitochondrial biomarkers and development of other adverse clinical outcomes were performed (Table 5).

20 out of 25 studies assessed association of levels of mitochondrial biomarkers and risks of ARDS development or progression (Table 5). Eight studies (40%) reported a significant association between higher levels of mitochondrial biomarkers and the risk of developing ARDS or worsening of ARDS severity (6, 14, 15, 24, 27–29, 31). The biomarkers that indicated significant correlation with ARDS progression include; xanthine, hypoxanthine (29), N-pentane (24). Lipid peroxidation markers (31), NADH, NADH2 (27), sirtuin, mediators of oxidative phosphorylation (27), and mtDNA (6, 14, 15). Nine studies (45%) reported non-significant trend toward association between biomarker levels and risk of development or progression of ARDS (12, 13, 16–18, 20, 24, 30, 33). The biomarker are as follows: MDA (17, 24), glutathione (17), ascorbate, alpha-tocopherol (30), GSH, N-acetylcysteine (18), metabolite ion chromatograph (20), and mtDNA (12, 13, 16, 33). One study showed an opposing finding, with decreased biomarker levels in association with ARDS disease progression, reporting higher lactate levels in non-ARDS compare to ARDS (23).

### Meta-analysis

**FIGURE 2:**
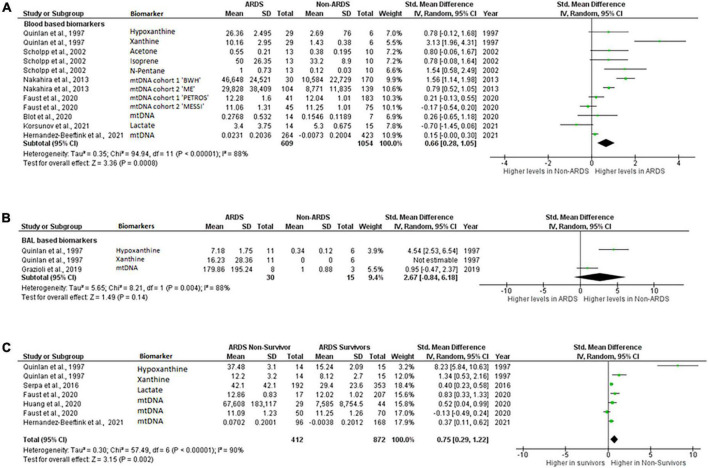
Levels of biomarkers of mitochondrial dysfunction. Forest plot meta-analysis of the levels of biomarkers in ARDS patients and non-ARDS controls in **(A)** blood samples *P* = 0.008 and **(B)** BAL samples *P* = 0.14 **(C)** ARDS survivors vs ARDS non-survivors, *P* = 0.002. Data analysis generated on RevMan 5.4.

Ten publications reported mean, standard deviation, and “*n*” number for inclusion in the meta-analysis. First, we compared the blood, plasma, broncho-alveolar lavage fluid (BAL), and lung epithelial lining fluid (ELF) levels of mitochondrial biomarkers between ARDS and non-ARDS subjects. To reflect the biological differences between biomarkers measured in the systemic circulation vs. lung compartment, peripheral blood, arterial blood and plasma were combined for comparison under the category “blood based biomarkers” and biomarkers measured in the BAL or ELF were combined under the category “BAL based biomarkers”. Eight out of ten studies were eligible for this comparison (6, 10, 13, 14, 16, 23, 24, 29). Several studies provided information on multiple biomarkers, Faust et al., and Nakahira et al., reported data from two cohorts, the data on different biomarkers and different cohorts were included in meta-analysis separately (Figure 2). Collectively, 609ARDS patients and 1,054 non-ARDS were included in the comparison, of these 743 ARDS and 1,363 non-ARDS samples were blood based and the remainder were BAL. Biomarkers that were eligible for meta-analysis inclusion were: xanthine, hypoxanthine, (29), acetone, N-pentane, isoprene (24), lactate (23) and mtDNA (6, 10, 13, 14, 16). Xanthine and hypoxanthine are mediators involved in mitochondrial redox balance (34), acetone, isoprene and N-pentane are indicators of metabolic changes in association with oxidative stress (35–37) and accumulation of lactate is a metabolic indicator of oxidative phosphorylation impairment (38).

Mitochondrial biomarker levels in the blood based samples were significantly higher in ARDS than in non-ARDS controls. Standardized mean difference 0.66 [0.28,1.05], overall effect *Z* = 3.36, *P* = 0.0008. Heterogeneity, *I*^2^ = 88%, *P* < 0.00001. *I*^2^ values show very large heterogeneity across non-ARDS and ARDS comparisons (Figure 2A).

Difference in the levels of the BAL based mitochondrial biomarkers did not reach statistical significance. Standardized mean difference 2.67 [–0.84,6.18], overall effect *Z* = 1.49, *P* = 0.14. Heterogeneity, *I*^2^ = 88%, *P* = 0.004. *I*^2^ values also show very large heterogeneity across non-ARDS and ARDS BAL biomarker comparisons (Figure 2B).

Next, levels of blood based mitochondrial biomarkers were compared between survivors and those who died from ARDS. The blood biomarkers eligible for this analysis were, hypoxanthine, xanthine (31), mtDNA (6, 15, 16) and lactate (22). All these biomarkers were measured within 24-h of enrollment. Mortality was recorded at 7- (15), 28- (22) or 30-days (6), Quinlan et al., did not specify the time when mortality was recorded (31) (Table 4). By meta-analysis, levels of biomarkers were significantly higher in non-survivors compared to survivors of ARDS. Standardized mean difference 0.37 [0.11,0.62], overall effect *Z* = 3.15, *P* = 0.002. Heterogeneity, *I*^2^ = 90%, *P* < 0.00001. *I*^2^ values show very large heterogeneity across non-ARDS and ARDS comparisons (Figure 2C).

**FIGURE 3:**
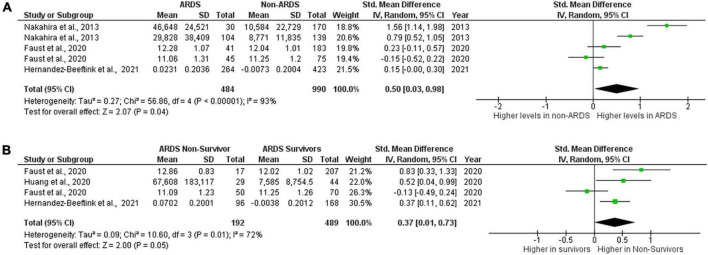
Levels of circulating mtDNA. Forest plot meta-analysis of the levels of circulating mtDNA in ARDS patients and non-ARDS controls, *P* = 0.04 **(A)** and in ARDS survivors vs ARDS non- survivors *P* = 0.05 **(B)**. Data analysis generated on RevMan 5.4.

Among the studies included in this review, mitochondrial DNA was the most frequently measured biomarker, therefore separate meta-analysis was performed on these studies. Three studies reported levels of mtDNA in plasma, serum or whole blood from ARDS and non-ARDS subjects (6, 10, 16). In the study of Nakahira et al., samples were collected upon enrollment into trial, Faust et al., collected samples upon arrival to emergency department (6, 10) and Hernández-Beeftink et al., recorded collection at 24 h after sepsis diagnosis (16). 484 ARDS patients and 990 non-ARDS patients were included in this comparison. Circulating mitochondrial DNA levels in patients with ARDS were significantly higher than in non-ARDS control groups. 0.50 [0.03,0.98], overall effect *Z* = 2.07, *P* = 0.04. Heterogeneity, *I*^2^ = 93%, *P* < 0.00001. Again, *I*^2^ values show very large heterogeneity across non-ARDS and ARDS comparisons (Figure 3A). Although Blot et al., and Grazioli et al., reported significant elevation of mtDNA in the BAL samples of ARDS patients compared to healthy controls in small cohorts (5 ARDS vs.3 heathy and 7 ARDS vs. 3 healthy, respectively), numerical information provided in these studies was not sufficient to carry out meta-analysis. Also, Nakahira et al., displayed graphs with significant differences in mtDNA copy numbers between patients with and without ARDS however raw values were not provided and thus could not be included in meta-analysis.

Three studies also reported data on the levels of circulating mtDNA in ARDS survivors and non-survivors (6, 15, 16). Both Haung et al., and Hernandez-Beeftink et al., collected samples at 24 h or 1 day after presentation (15, 16), Faust et al., collected samples at presentation (Table 3). There were 489 ARDS survivors and 192 ARDS non-survivors included in the comparison. Mortality was recorded at 30 days (Faust et al.), 28 days (Hernandez-Beeftink et al.) and 7 days (Huang et al.) (Table 4). Results of meta-analysis did not allow to draw definitive conclusions about whether or not levels of mtDNA are elevated in non-survivors as overall *P* value is on the border of significance, although there is a numeric trend toward higher levels in non-survivors. Standardized mean difference 0.37 [0.01,0.73], overall effect *Z* = 2.00, *P* = 0.05. Heterogeneity, *I*^2^ = 72%, *P* = 0.01. *I*^2^ values show large heterogeneity across ARDS survivor and non-survivor comparisons (Figure 3B).

### Publication bias

**FIGURE 4:**
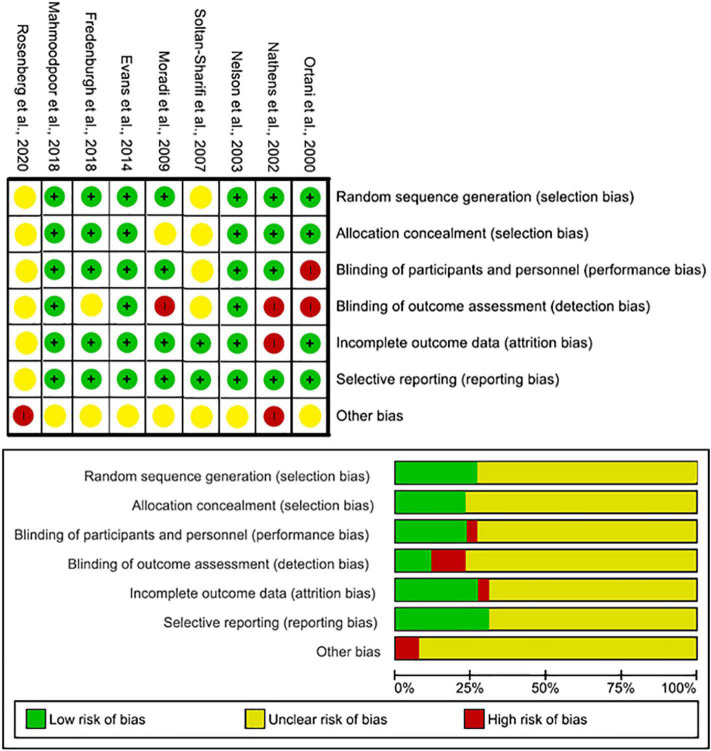
Quality assessment Cochrane risk of bias for RCT trial studies.

The nine RCT trials were assessed using the Cochrane risk of bias charts. Five studies were classed as low risk, and two as high risks (Figure 4 and Table 6) (17–21, 30–33). The remaining 16, cohort and case–control studies, were assessed by the Newcastle–Ottawa Scale (NOS) (10, 13–16, 20, 22–29). The mean score was 6. 12 out of 16 of studies were considered low risk (Table 7).

**TABLE 6:** Quality assessment Cochrane risk of bias table justifications for RCT trial studies.

	Random sequence generation (selection bias)	Allocation concealment (selection bias)	Blinding of participants and personnel (performance bias)	Blinding of outcome assessment (detection bias)	Incomplete outcome data (attrition bias)	Selective reporting (reporting bias)	Other bias
Ortolani et al. (17)	Random assignment of participants into treatment groups		Non-blinded study		Evenly assigned patient groups, no loose of patients in follow up	All outcome data reported	Other bias not discussed
Nathens et al. (30)	Random assignment of participants into treatment groups (1:1)		Personnel were blinded to grouping [carried out by computer and Pharmacy (non-investigators)]. Participants were non-blinded, but data taken from samples collected from blood thus removing this concern	Non-blinded for ease of care organization	High level of exclusion post trial administration	All outcome data reported	Lack of placebo for control group of inte rest for this SR and blinding for investigators post group assignment.
Nelson et al. (31)	Double blinded, controlled, randomized multicenter trial, permuted-block randomization design				Full patient follow up	All outcome data reported	Other bias not discussed
Soltan-Sharifi et al. (18)	Information not disclosed	Information not disclosed	Information not disclosed	Information not disclosed	Full patient follow up	All outcome data reported	Other bias not discussed
Moradi et al. (19)	Simple randomization was performed	Information not disclosed	Single blinding	Non-blinded	Full patient follow up	All outcomes set out, were recorded	Other bias not discussed
Evans et al. (20)	Randomized patients into treatment to control	Samples random order and were assigned to a random LC-MS run order using a computerized algorithm.	Agilent MassHunter Quantitative Analysis software, with the analyst blinded to the identity of the subjects.		Full patient follow up	All outcomes set out, were recorded	Other bias not discussed
Fredenburgh et al. (33)	Phase one unmasked for safety reasons, phase two was masked. Data taken only from phase two in relation for this SR.	Random allocation hidden from trial executives	Masking of which group participants where in as well as	Unclear	Full patient follow up	Data, even if unsuccessful reported	Other bias not discussed
Mahmoodpoor et al. (21)	Random assignment of participants into treatment groups (1:1)		Masking of which group participants where in as well as	Double blinded study	Full patient follow up	Data, even if unsuccessful reported	Other bias not discussed
Rosenberg et al. (32)	Information not disclosed	Information not disclosed	Information not disclosed	Information not disclosed	Information not disclosed	Information not disclosed	Potential recall bias

Risk of bias is higher with greater intensity of gray.

**TABLE 7:** Quality assessment Newcastle-Ottawa scale table for non-RCT studies.

Studies	Represen-tativeness of the exposed cohort	Selection of the non-exposed cohort	Ascertain-ment of exposure	Demon-stration that outcome of interest was not present at start of study	Compa-rability of cohorts on the basis of the design or analysis	Assessment of outcome	Was follow-up long enough for outcomes to occur	Adequacy of follow up of cohorts	Total
Quinlan et al. (29)	*	*	NR	*	*	*	NR	*	6
Scholpp et al. (24)	*	*	*	*	*	*	*	*	8
Nakahira et al. (10)	*	*	*	*	*	*	*	*	8
Bhargava et al. (26)	*	*	*	NA	*	*	*	NA	6
Evans et al. (20)	*		*	*					3
Liu et al. (25)	*		NR	*	*	*		*	5
Serpa et al. (22)	*	*	NR	*	*	*	*	*	7
Dorward et al. (27)	*	*	*	*	*	*	*	*	8
Garramone et al. (11)		*	NR	*		*	*		4
Grazioli et al. (13)		*	NR	*		*			3
Bos et al. (28)	*	*	Unclear	*	*	*	*	*	7
Blot et al. (14)		*	*	*	*	*	*	*	7
Huang et al. (15)	*	*	*	*	*	*	*	*	8
Faust et al. (6)	*	*	*	*	*	*	*	*	8
Korsunov et al. (23)		*	NR	*	*	*			4
Hernández -Beeftink et al. (16)	*	*	*	*	*	*			6

NR, not recorded. *One score.

## Discussion

Clinical and biological markers for prediction of ARDS outcomes are based upon inflammatory indicators, including IL-6, IL-8, RAGE, Ang-2, C-reactive protein and procalcitonin (39, 40). A systematic review by van der Zee et al. examined the multivariate biomarkers associated with ARDS disease, in which RAGE and Ang-2 showed significant association with the risk of ARDS development; yet none were significantly correlated to mortality (40). This likely is contingent on the heterogeneous nature of ARDS pathophysiology.

Pneumonia and sepsis were the top two the most frequent and most devastating causes of ARDS in the studies included in the review. Recently mitochondrial dysfunction, specifically ability of immune cells to switch between glycolytic and oxidative phosphorylation pathways has emerged as a mechanism of pathogenesis of sepsis (41). Patients with sepsis have been shown to have decreased expression of mitochondrial quality mitophagy markers PINK1 and PARKIN, elevated levels of mtDNA, dysfunctional mitochondrial morphology and decreased mitochondrial mass, as well as increased cell death due to calcium overload and raised levels of reactive oxygen species (42–44). The consolidated contribution of mitochondrial dysfunction with the pathogenesis of sepsis, alongside the well-established sepsis induction of ARDS; combined made it plausible to hypothesize that mitochondrial dysfunction might too contribute to ARDS (5, 45).

To our knowledge, this is the first systematic review with meta-analysis investigating association of levels of biomarkers of mitochondrial dysfunction with ARDS. Majority of studies included into this review reported positive trends toward association of elevated levels of biomarkers of mitochondria dysfunction with ARDS. These trends reached statistical significance in the cases of mtDNA, xanthine, hypoxanthine, lactate, isoprene and *n*-pentane in the blood based samples, however statistically significant difference is absent in BAL samples. Of note, levels of xanthine were not detectable in the BAL of non-ARDS patients which could have impacted the results of meta-analysis.

Importantly, levels of circulating hypoxanthine, xanthine, mtDNA and lactate measured at early time points after presentation were significantly elevated in those who survived ARDS compared to non-survivors, suggesting a potential role of mitochondrial dysfunction in ARDS pathogenesis.

MtDNA was the most frequently measured biomarker across the included studies. Levels of circulating cell-free mtDNA were significantly higher in ARDS patients compared to non-ARDS in six studies. This was further confirmed by meta-analysis of the three studies which have provided necessary raw values for comparison. Difference in mtDNA levels between ARDS survivors and non-survivors did not reach statistical significance by meta-analysis with overall *P* = 0.05, however there was strong trend toward higher levels in non-survivors. Interestingly, Faust et al., also reported mtDNA levels at 48 h after presentation which were significantly higher in non-survivors than in survivors in both cohorts (6), suggesting that later time points might be more appropriate for measurements of mtDNA as predictor of mortality in ARDS. Taken together, these data indicate an association of mitochondrial dysfunction with ARDS pathophysiology and highlight blood mtDNA as important mediator of ARDS pathogenesis with the potential to serve as a biomarker for predicting the risk of mortality.

This systematic review identified ten different biomarkers of mitochondrial dysfunction measured in ARDS patients. Initial overview of mitochondrial biomarkers in ARDS vs non-ARDS patients showed significantly higher levels in the ARDS patient groups, regardless of the cause of ARDS (Figure 2 and Tables 4, 5). However, 6 of these biomarkers were only measured in one study; the top four most frequently measured biomarkers were (i) mtDNA, (ii) glutathione, (iii) lactate, and (iv) MDA. The study weighting of the meta-analysis was largely driven by blood mtDNA as the most frequently measured biomarker. Therefore, we carried out separate meta-analysis of the studies that reported levels of mtDNA. Plasma mtDNA levels were significantly higher in ARDS vs non-ARDS at time points from 0 to 24 h from presentation. Interestingly, Bolt et al., and Grazioli et al., also reported significant elevation in mtDNA levels in the BAL samples in ARDS patients compared to non-ARDS controls, although the information provided in these studies was not sufficient to run meta-analysis. However, the sample size was small in both studies (7 heathy vs. 7 ARDS and 3 healthy vs. 5ARDS BAL samples, respectively), therefore further studies are required to investigate the significance of alveolar release of mtDNA in ARDS, as a potential biomarker of lung injury.

Mitochondrial DNA levels are currently used as prognostic biomarker in a number of diseases such as Parkinson’s disease and type two diabetes in combination with coronary heart disease (46). Hernandez-Beeftink et al., observed that mtDNA copies in the whole blood were significantly associated with 28-day survival in sepsis patients who developed ARDS (hazard ratio = 3.65, 95% confidence interval = 1.39–9.59, *p* = 0.009) but not in sepsis patients without ARDS. These findings support the hypothesis that cell free mtDNA copies at sepsis diagnosis could be considered an early prognostic biomarker in sepsis-associated ARDS patients. Results of this review support the potential use of mtDNA as ARDS biomarker; however, more research would be required to determine the most appropriate sample (plasma or whole blood) as well as best time points for sample collection. Additionally, studies recorded mtDNA levels in different units (e.g., copy numbers per μl, μmol, intensities); although the meta-analysis model considers this factor, there is a need for standardization of the measurement units. Circulating mtDNA levels may facilitate the stratification of patients, however, future studies are necessary to standardize the technique and to define more accurate cut-off points.

Glutathione, and its downstream mediators, hypoxanthine and xanthine, follow a similar trend to mtDNA, with elevated levels in ARDS patients (Tables 1, 2). Both hypoxanthine and xanthine are converted to uric acid through xanthine oxidase, resulting in ROS production and leading to oxidative stress (47, 48). In similar fashion, decreased glutathione reduction and increased redox imbalances are known to be associated with mitochondrial disorders (49). It is feasible that one, or both of these mediators could act as prognostic biomarkers for ARDS given the positive trends observed across the two studies (20, 21). Glutathione mediators were recorded in the blood and BAL samples, demonstrating similar trends. To further this avenue, measurement of all three ROS mediators in larger cohorts would be required.

Oxidative damage to lipids, amino acids, and DNA leads to accumulation of malondialdehyde (MDA) (Tables 4, 5). MDA inhibits mitochondrial complex I, II and V, thus impacting the functionality of present mitochondria (50). Due to lack of control comparator for mortality/other worse outcomes, it was not possible to draw any definitive conclusions in regards to MDA. One out of three studies found significantly higher levels in ARDS compared to non-ARDS (17, 51), while the other two studies showed a non-significant trend with higher levels in ARDS (24). MDA was measured in both BAL and plasma; this variation could be driving the lack of conclusive results. Interestingly, studies which investigated the levels of lactate, another metabolite indirectly representative of mitochondrial dysfunction, also reported controversial findings (52). Evans et al., observed a fold-change increase in lactate in ARDS vs non-ARDS, while Serpa et al., demonstrated a higher level of lactate in ARDS non-survivors. On the contrary, Korsunov et al., reported higher levels of lactate in non-ARDS vs ARDS. No data comparisons around lactate were significant across the three studies (20, 22, 23). As there is no consistency across all three studies, it is plausible that metabolites such as MDA and lactate are not useful as prospective biomarkers for ARDS clinical outcomes (53).

One study examined a biomarker associated to mitochondria genetic defects; GDF-15 (Tables 4, 5). GDF-15 is a secretory protein induced by mitochondrial stress, overexpressed in patients with mitochondrial point mutation syndromes (54, 55). The outcomes of this biomarker, as described in Tables 4, 5, do indicate a possible link of association of mitochondrial dysfunction with ARDS, however the lack of raw numbers and limited size of the cohort did not allow for definitive conclusions; current evidence would not support the use of this genetic biomarker in ARDS.

The quality assessment imply a minimal risk of bias across the board of studies included. Main findings were drawn from meta-analysis, of which only one study, Korsunov et al., presented with NOS score of four, due to lack of provided information.

## Limitations

This review had several limitations. First, the lack of global representation across study cohorts could influence the predictive power of the examined biomarkers. Secondly, a large variance in study size resulted in high *I*^2^ values across all meta-analysis carried out; some of the smaller studies included less than 100 patients could be underpowered for significance calculations. Finally, regardless of standardized mean difference calculation weighting of studies, the inconsistency in biomarker units, as well as different methods of analysis of the same type of biomarkers could affect the statistical conclusions drawn from this small-scale meta-analysis.

## Conclusion

This systematic review and meta-analysis suggest that increased levels of biomarkers of mitochondrial dysfunction are positively associated with ARDS. Blood-based biomarkers were the most appropriate for assessment of mitochondrial dysfunction. Circulating mtDNA is the most frequently measured biomarker of mitochondrial dysfunction; circulating mtDNA levels are significantly higher in ARDS patients compared to non-ARDS controls. Mitochondrial DNA is a plausible biomarker candidate for further investigation of its role in ARDS pathogenesis. Further research is required to explore the role of mitochondrial biomarkers in greater populations of ARDS patients and between ARDS subphenotypes.

## Data availability statement

The datasets presented in this study can be found in online repositories. The names of the repository/repositories and accession number(s) can be found in the article/Supplementary material.

## Author contributions

AK designed the study, revised and independently checked the manuscript. CM and NM conducted the systematic searches and data extraction. CM merged the studies and wrote the manuscript. All authors approved the submitted version.

## Footnotes

1
https://www.sign.ac.uk/assets/study_design.pdf

2
https://methods.cochrane.org/bias/resources/rob-2-revised-cochrane-risk-bias-tool-randomized-trials

3
http://www.ohri.ca/programs/clinical_epidemiology/oxford.asp

## References

[1] Sweeney RM McAuley D. Acute respiratory distress syndrome. *Lancet.* (2016) 388:2416–30. 27133972 10.1016/S0140-6736(16)00578-X PMC7138018

[2] Matthay M Zemans R Zimmerman G Arabi Y Beitler J Mercat A Acute respiratory distress syndrome. *Nat Rev Dis Prim.* (2018) 5:18. 10.1038/s41572-019-0069-0 30872586 PMC6709677

[3] Fan E Beitler J Brochard L Calfee C Ferguson N Slutsky A COVID-19-associated acute respiratory distress syndrome: is a different approach to management warranted? *Lancet Respir Med.* (2020) 8:816–21. 10.1016/S2213-2600(20)30304-0 32645311 PMC7338016

[4] Schumacker P Gillespie M Nakahira K Choi A Crouser E Piantadosi C Mitochondria in lung biology and pathology: more than just a powerhouse. *Am J Physiol Lung Cell Mol Physiol.* (2014) 306:962–74. 10.1152/ajplung.00073.2014 24748601 PMC4042189

[5] Ten V Ratner V. Mitochondrial bioenergetics and pulmonary dysfunction: current progress and future directions. *Paediatr Respir Rev.* (2020) 34:37–45. 10.1016/j.prrv.2019.04.001 31060947 PMC6790157

[6] Faust H Reilly J Anderson B Ittner C Forker C Zhang P Plasma mitochondrial DNA levels are associated with ARDS in trauma and sepsis patients. *Chest.* (2020) 157:67–76. 10.1016/j.chest.2019.09.028 31622590 PMC6965693

[7] Simmons J Lee Y Pastukh V Capley G Muscat C Muscat D Potential contribution of mitochondrial (mt) DNA damage associated molecular patterns (DAMPs) in transfusion products to the development of acute respiratory distress syndrome (ARDS) after multiple transfusions. *Physiol Behav.* (2017) 176:139–48. 10.1097/TA.0000000000001421 28301393 PMC5472063

[8] Rossignol D Frye R. Mitochondrial dysfunction in autism spectrum disorders: a systematic review and meta-analysis. *Mol Psychiatry.* (2012) 17:290–314. 10.1038/mp.2010.136 21263444 PMC3285768

[9] Cloonan S Kim K Esteves P Trian T Barnes P. Mitochondrial dysfunction in lung ageing and disease. *Eur Respir Rev.* (2020) 29:200165. 10.1183/16000617.0165-2020 PMC9488551 33060165

[10] Nakahira K Kyung S Rogers A Gazourian L Youn S Massaro A Circulating mitochondrial DNA in patients in the ICU as a marker of mortality: derivation and validation. *PLoS Med.* (2013) 10:e1001577; discussione1001577. 10.1371/journal.pmed.1001577 24391478 PMC3876981

[11] Garramone A Cangemi R Bresciani E Carnevale R Bartimoccia S Fante E Early decrease of oxidative stress by non-invasive ventilation in patients with acute respiratory failure. *Intern Emerg Med.* (2018) 13:183–90. 10.1007/s11739-017-1750-5 28914417

[12] Pan X Wang L Fei G Dong J Zhong C Lu J Acute respiratory failure is the initial manifestation in the adult-onset A3243G tRNALeu mtDNA mutation: a case report and the literature review. *Front Neurol.* (2019) 10:780. 10.3389/fneur.2019.00780 31379729 PMC6657224

[13] Grazioli S Dunn-Siegrist I Pauchard L Blot M Charles P Pugin J. Mitochondrial alarmins are tissue mediators of ventilator-induced lung injury and ARDS. *PLoS One.* (2019) 14:e0225468. 10.1371/journal.pone.0225468 31756204 PMC6874419

[14] Blot M Jacquier M Aho Glele L Beltramo G Nguyen M Bonniaud P CXCL10 could drive longer duration of mechanical ventilation during COVID-19 ARDS. *Crit Care.* (2020) 24:632. 10.1186/s13054-020-03328-0 PMC7604548 33138839

[15] Huang L Chang W Huang Y Xu X Yang Y Qiu H. Prognostic value of plasma mitochondrial DNA in acute respiratory distress syndrome (ARDS): a single-center observational study. *J Thorac Dis.* (2020) 12:1320–8. 10.21037/jtd.2020.02.49 32395269 PMC7212167

[16] Hernández-Beeftink T Guillen-Guio B Rodríguez-Pérez H Marcelino-Rodríguez I Lorenzo-Salazar J Corrales A Whole-blood mitochondrial DNA copies are associated with the prognosis of acute respiratory distress syndrome after sepsis. *Front Immunol.* (2021) 12:737369. 10.3389/fimmu.2021.737369 34557198 PMC8453061

[17] Ortolani O Conti A De Gaudio A Masoni M Novelli G. Protective effects of N-Acetylcysteine and rutin on lipid peroxidation of the lung epithelium during the adult respiratory distress syndrome. *Shock.* (2000) 13:14–8. 10.1097/00024382-200013010-00003 10638663

[18] Soltan-Sharifi M Mojtahedzadeh M Najafi A Khajavi M Rouini M Moradi M Improvement by N-acetylcysteine of acute respiratory distress syndrome through increasing intracellular glutathione, and extracellular thiol molecules and anti-oxidant power: evidence for underlying toxicological mechanisms. *Hum Exp Toxicol.* (2007) 26:697–703. 10.1177/0960327107083452 17984140

[19] Moradi M Mojtahedzadeh M Mandegari A Soltan-Sharifi M Najafi A Khajavi M The role of glutathione-S-transferase polymorphisms on clinical outcome of ALI/ARDS patient treated with N-acetylcysteine. *Respir Med.* (2009) 103:434–41. 10.1016/j.rmed.2008.09.013 18993042

[20] Evans C Karnovsky A Kovach M Standiford T Burant C Stringer K. Untargeted LC-MS metabolomics of bronchoalveolar lavage fluid differentiates acute respiratory distress syndrome from health. *J Proteome Res.* (2014) 13:640–9. 10.1021/pr4007624 24289193 PMC4068805

[21] Mahmoodpoor A Hamishehkar H Shadvar K Ostadi Z Sanaie S Saghaleini S The effect of intravenous selenium on oxidative stress in critically ill patients with acute respiratory distress syndrome. *Immunol Invest.* (2019) 48:147–59. 10.1080/08820139.2018.1496098 30001171

[22] Serpa Neto A Schmidt M Azevedo L Bein T Brochard L Beutel G Associations between ventilator settings during extracorporeal membrane oxygenation for refractory hypoxemia and outcome in patients with acute respiratory distress syndrome: a pooled individual patient data analysis: mechanical ventilation during ECMO. *Intens Care Med.* (2016) 42:1672–84. 27586996 10.1007/s00134-016-4507-0 PMC7094949

[23] Korsunov V Georgiyants M Skoryk V. Central hemodynamics and oxygen transport in patients with acute respiratory distress syndrome caused by Covid-19 and their impact on the course and outcomes of the disease. *EUREKA Heal Sci.* (2021) 1:3–11.

[24] Scholpp J Schubert J Miekisch W Geiger K. Breath markers and soluble lipid peroxidation markers in critically III patients. *Clin Chem Lab Med.* (2002) 40:587–94. 10.1515/CCLM.2002.101 12211653

[25] Liu D Luo G Luo C Wang T Sun G Hei Z. Changes in the concentrations of mediators of inflammation and oxidative stress in exhaled breath condensate during liver transplantation and their relations with postoperative ARDS. *Respir Care.* (2015) 60:679–88. 10.4187/respcare.03311 25628453

[26] Bhargava M Becker T Viken K Jagtap P Dey S Steinbach M Proteomic profiles in acute respiratory distress syndrome differentiates survivors from non-survivors. *PLoS One.* (2014) 9:e109713. 10.1371/journal.pone.0109713 25290099 PMC4188744

[27] Dorward D Lucas C Doherty M Chapman G Scholefield E Conway Morris A Novel role for endogenous mitochondrial formylated peptide-driven formyl peptide receptor 1 signalling in acute respiratory distress syndrome. *Thorax.* (2017) 72:928–36. 10.1136/thoraxjnl-2017-210030 28469031 PMC5738532

[28] Bos L Scicluna B Ong D Cremer O Van Der Poll T Schultz M. Understanding heterogeneity in biologic phenotypes of acute respiratory distress syndrome by leukocyte expression profiles. *Am J Respir Crit Care Med.* (2019) 200:42–50. 30645145 10.1164/rccm.201809-1808OC

[29] Quinlan G Lamb N Tilley R Evans T Gutteridge J. Plasma hypoxanthine levels in ARDS: implications for oxidative stress, morbidity, and mortality. *Am J Respir Crit Care Med.* (1997) 155:479–84. 10.1164/ajrccm.155.2.9032182 9032182

[30] Nathens A Neff M Jurkovich G Klotz P Farver K Ruzinski J Randomized, prospective trial of antioxidant supplementation in critically III surgical patients. *Ann Surg.* (2002) 236:814–22. 10.2174/138955707781024526 12454520 PMC1422648

[31] Nelson J DeMichele S Pacht E Wennberg A Gadek J Drake J Effect of enteral feeding with eicosapentaenoic acid, γ-linolenic acid, and antioxidants on antioxidant status in patients with acute respiratory distress syndrome. *J Parenter Enter Nutr.* (2003) 27:98–104. 10.1177/014860710302700298 12665164

[32] Rosenberg B Hirano M Quinzii C Colantuoni E Needham D Lederer D Growth differentiation factor-15 as a biomarker of strength and recovery in survivors of acute respiratory failure. *Thorax.* (2019) 74:1099–101. 10.1136/thoraxjnl-2019-213621 31534031 PMC7043788

[33] Fredenburgh L Perrella M Barragan-Bradford D Hess D Peters E Welty-Wolf K A phase I trial of low-dose inhaled carbon monoxide in sepsis-induced ARDS. *JCI Insight.* (2018) 3:e124039. 10.1172/jci.insight.124039 PMC6328240 30518685

[34] Gladden J Zelickson B Wei C Ulasova E Zheng J Ahmed M Novel insights into interactions between mitochondria and xanthine oxidase in acute cardiac volume overload. *Free Radic Biol Med.* (2011) 51:1975–84. 10.1016/j.freeradbiomed.2011.08.022 21925594 PMC3364106

[35] Tanda N Hoshikawa Y Sato T Takahashi N Koseki T. Exhaled acetone and isoprene in perioperative lung cancer patients under intensive oral care: possible indicators of inflammatory responses a nd metabolic changes. *Biomed Res.* (2019) 40:29–36. 10.2220/biomedres.40.29 30787261

[36] Hall J Crane F. Disruption of mitochondrial membrane by acetone extraction. *Biochim Biophys Acta.* (1971) 2:682–6. 10.1016/0005-2736(71)90067-8 5159802

[37] Akobsson-Borin A Aberg F Dallner G. Lipid peroxidation of microsomal and mitochondrial membranes extracted with n-pentane and reconstituted with ubiquinol, dolichol and cholesterol. *Biochim Biophys Acta.* (1994) 2:159–66. 10.1016/0005-2760(94)90022-1 8025126

[38] Villar J Herrán-Monge R González-Higueras E Prieto-González M Ambrós A Rodríguez-Pérez A Clinical and biological markers for predicting ARDS and outcome in septic patients. *Sci Rep.* (2021) 11:22702. 10.1038/s41598-021-02100-w PMC8608812 34811434

[39] Yan J Rao Q. Biomarkers in the diagnosis and prognostic assessment of acute respiratory distress syndrome. *J Transl Intern Med.* (2014) 2:160–3.

[40] Van Der Zee P Rietdijk W Somhorst P Endeman H Gommers D. A systematic review of biomarkers multivariately associated with acute respiratory distress syndrome development and mortality. *Crit Care.* (2020) 24:243. 10.1186/s13054-020-02913-7 32448370 PMC7245629

[41] Rahmel T Marko B Nowak H Bergmann L Thon P Rump K Mitochondrial dysfunction in sepsis is associated with diminished intramitochondrial TFAM despite its increased cellular expression. *Sci Rep.* (2020) 10:21029. 10.1038/s41598-020-78195-4 33273525 PMC7713186

[42] Knutie S Gabor C Kohl K Rohr J. Mitochondrial DNA in Sepsis. *Physiol Behav.* (2017) 176:139–48.28363838

[43] van der Slikke E Star B van Meurs M Henning R Moser J Bouma H. Sepsis is associated with mitochondrial DNA damage and a reduced mitochondrial mass in the kidney of patients with sepsis-AKI. *Crit Care.* (2021) 25:36. 10.1186/s13054-020-03424-1 33494815 PMC7831178

[44] Preau S Vodovar D Jung B Lancel S Zafrani L Flatres A Energetic dysfunction in sepsis: a narrative review. *Ann Intens Care.* (2021) 11:104. 10.1186/s13613-021-00893-7 34216304 PMC8254847

[45] Robinson MJ Krasnodembskaya AD. Therapeutic targeting of metabolic alterations in acute respiratory distress syndrome. *Eur Respir Rev*. (2020) 29:200114. 10.1183/16000617.0114-2020 32620587 PMC9488567

[46] Lowes H Pyle A Santibanez-Koref M Hudson G. Circulating cell-free mitochondrial DNA levels in Parkinson’s disease are influenced by treatment. *Mol Neurodegener.* (2020) 15:10. 10.1186/s13024-020-00362-y 32070373 PMC7029508

[47] Kristal B Vigneau-Callahan K Moskowitz A Matson W. Purine catabolism: links to mitochondrial respiration and antioxidant defenses? *Arch Biochem Biophys.* (1999) 370:22–33. 10496973 10.1006/abbi.1999.1387

[48] Vergeade A Mulder P Vendeville C Ventura-Clapier R Thuillez C Monteil C. Xanthine oxidase contributes to mitochondrial ROS generation in an experimental model of cocaine-induced diastolic dysfunction. *J Cardiovasc Pharmacol.* (2012) 60:538–43. 10.1097/FJC.0b013e318271223c 22967988

[49] Enns G Cowan T. Glutathione as a redox biomarker in mitochondrial disease—implications for therapy. *J Clin Med.* (2017) 6:50. 10.3390/jcm6050050 28467362 PMC5447941

[50] Long J Liu C Sun L Gao H Liu J. Neuronal mitochondrial toxicity of malondialdehyde: inhibitory effects on respiratory function and enzyme activities in rat brain mitochondria. *Neurochem Res.* (2009) 34:786–94. 10.1007/s11064-008-9882-7 19023656

[51] Liu J Zou Y Tang Y Xi M Xie L Zhang Q Circulating cell-free mitochondrial deoxyribonucleic acid is increased in coronary heart disease patients with diabetes mellitus. *J Diabetes Investig.* (2016) 7:109–14. 10.1111/jdi.12366 26816608 PMC4718102

[52] Glancy B Kane D Kavazis A Goodwin M Willis W Gladden L. Mitochondrial lactate metabolism: history and implications for exercise and disease. *J Physiol.* (2021) 599:863–88. 32358865 10.1113/JP278930 PMC8439166

[53] Metwaly S Winston B. Systems biology ARDS research with a focus on metabolomics. *Metabolites.* (2020) 10:207. 10.3390/metabo10050207 32438561 PMC7281154

[54] Ayuso P Martínez C Pastor P Lorenzo-Betancor O Luengo A Jiménez-Jiménez F An association study between heme oxygenase-1 genetic variants and Parkinson’s disease. *Front Cell Neurosci.* (2014) 8:298. 10.3389/fncel.2014.00298 25309329 PMC4173932

[55] Fujita Y Ito M Ohsawa I. Mitochondrial stress and GDF15 in the pathophysiology of sepsis. *Arch Biochem Biophys.* (2020) 696:108668. 10.1016/j.abb.2020.108668 33188737

