# Supplementary material for: Biomarkers of mitochondrial dysfunction in acute respiratory distress syndrome: A systematic review and meta-analysis
Source: Front Med (Lausanne). 2022 Dec 14;9:1011819. doi: 10.3389/fmed.2022.1011819 (PMC9795057; doi:10.3389/fmed.2022.1011819)
Supplement: Supplementary file 1 [file Data_Sheet_1.pdf]

Supplementary Material

1 Supplementary Methods

Study Eligibility criteria

In order to be eligible studies must include adult (18y/o) participants with ARDS in intensive care units (ICU) (critically ill patients). The severity, cause, and duration of ARDS will not be restricted. The definition of ARDS will not be a limiting factor. Covid-ARDS will not be included in this systematic review due to differences in disease pathophysiology. No other exclusion criteria will be applied to patients.

Eligibility criterion was applied to search engines through limitation and exclusion filters, based upon presence/ lack of terms in title or abstract of article. Studies included were restricted to peer-reviewed full-text accessible original/ primary articles in English. Based upon earliest article available, the cut off points for inclusion were 1967 to 2021. All study designs will be eligible for inclusion in this systematic review. Studies lacking a comparator/control, for example in the instance of retrospective case reports, were not eligible for meta-analysis inclusion. In the case of interventional studies, the data were extracted from the non-interventional/control arm of the study; this was to ensure that the mitochondrial biomarkers were not confounded by the intervention carried out.

Furthermore, articles were excluded if any of the following criteria applied: (i) research conducted in vitro/ in vivo; (ii) review papers; (iii) conference proceedings; (iv) articles without a minimum of one criterion from each search strategy column (Table.1). Any disagreement regarding study eligibility was resolved by Dr Krasnodembskaya.

Outcome data collection process and analysis

Data was collected by McClintock and Mulholland independently from all studies which satisfied the selection criterion, and later reassessed by all included authors. In cases where exact data values were not provided in full text studies or supplementary information, corresponding authors for each study were contacted. Those who did not supply requested data information were omitted from the meta-analysis portion of this systematic review.
